# Medial unicondylar knee arthroplasty should be reserved for patients with complete joint space collapse

**DOI:** 10.1007/s00167-021-06588-7

**Published:** 2021-05-01

**Authors:** Alexander Wurm, Anna Zechling, Hermann Leitner, Dietmar Dammerer, Bernhard Pfeifer, Martin Krismer, Michael Liebensteiner

**Affiliations:** 1 Department of Orthopaedics and Traumatology, Medical University of Innsbruck, Anichstrase 35, 6020 Innsbruck, Austria; 2 Medical University of Innsbruck, Innsbruck, Austria; 3 Department of Clinical Epidemiology, Tyrolean Federal Institute for Integrated Care, Tirol Kliniken GmbH, Innsbruck, Austria

**Keywords:** Total knee arthroplasty, Joint space narrowing, Joint space width, Osteoarthritis, Total knee replacement, Outcome

## Abstract

**Purpose:**

To determine whether preoperative radiologic joint space width (JSW) is related to the outcome of medial unicondylar knee arthroplasty (UKA) (primary hypothesis).

**Methods:**

A retrospective comparative analysis was performed. One group was comprised of UKA patients with preoperative JSW 0–1 mm. Another group was made up of patients with preoperative JSW ≥ 2 mm (range 0–4 mm). The JSW was measured from preoperative weight-bearing Schuss-view radiographs. The clinical outcome was determined with the Western Ontario and MacMaster Universities (WOMAC) Osteoarthritis Index score preoperatively and 1 year after medial UKA. Implant survival data were obtained from the arthroplasty register of Tyrol.

**Results:**

There were 80 patients with a preoperative JSW 0–1 mm (age 66, BMI 27.8) and 70 patients with a preoperative JSW ≥ 2 mm (age 64, IQR 15, BMI 28.1). WOMAC total was 10 ± 10 in patients with 0–1 mm JSW and 25 ± 47 in patients with ≥ 2 mm JSW at 1 year postoperative (*p* = 0.052). WOMAC pain at 1 year postoperative was 7 ± 16 in patients with 0–1 mm JSW and 18 ± 46 in patients with ≥ 2 mm JSW (*p *= 0.047). WOMAC function at 1 year postoperative was 10 ± 9 in patients with 0–1 mm JSW and 17 ± 51 in patients with ≥ 2 mm JSW (*p *= 0.048). In patients with 0–1 mm JSW 5 year prosthesis survival was 92.3% and in patients with ≥ 2 mm JSW, it was 81.1% (*p *= 0.016).

**Conclusions:**

In patients with preoperative complete joint space collapse (0–1 mm JSW), clinical outcome was superior to that of patients with incomplete joint space collapse. This was true for both 1 year postoperative WOMAC pain and WOMAC function and for 5 year implant survival rates. On the basis of our findings, it is recommended that ‘complete joint space collapse’ especially be used to achieve best clinical outcome in medial UKA surgery.

**Level of evidence:**

IV.

## Introduction

In the field of medial unicondylar knee arthroplasty (UKA), only three studies exist that examined the relationship between preoperative OA severity and clinical outcome [5, 7, 10]. Knifsund et al. investigated 294 UKA cases [7]. They reported that those undergoing UKA with preoperative Kellgren–Lawrence (KL) Grade 0–2 had a significantly greater risk for later reoperation than did those with KL Grade 3–4. Knifsund et al. also stated that knees with a joint space width of more than 1 mm have a greater risk for revision surgery. However, they did not report patient-reported outcome (e.g. knee scores). Maier et al. also investigated the influence of the preoperative stage of OA on UKA outcome [10]. The authors reported on 64 patients and compared cases with partial joint space collapse and those with complete joint space collapse regarding knee scores and implant survival. There were no significant differences in knee score outcome and also no significant differences in revision rates. Hamilton et al. analysed 94 UKA with preoperatively only partial cartilage loss [5]. Outcome in those patients was poorer than in patients with full-thickness cartilage loss: more reoperations, inferior results in Oxford Knee Score. In summary, studies of that topic were rare and the three available studies provided conflicting information.

In light of the shortcomings of previous research, our study approach incorporated: (a) robust implant survival data from the arthroplasty registry of Tyrol, (b) use of a patient-reported outcome score along with implant survival, (c) use of the OA staging method ‘joint space width’ that was reported to be superior to Kellgren–Lawrence stages.

It was hypothesised that patients without complete radiologic joint space collapse would experience a different clinical knee score outcome (WOMAC score) than would those with complete radiologic joint space collapse (primary hypothesis).

## Methods

A retrospective comparative design was applied. Data already available from clinical routine were analysed after approval by the Ethics Committee of the medical university (approval No. AN2017-0021-370/4.1). Analysed were patients who had previously undergone primary UKA at our department as part of clinical routine. Inclusion/exclusion of patients was handled in accordance with the Oxford surgical manual. In addition, patients were excluded from data analysis in the case of: (a) incomplete WOMAC data, (b) primary prostheses other than medial Oxford UKA, and (c) missing preoperative Schuss-view radiograph.

All surgical procedures were performed as part of our hospital’s clinical routine. Patients always underwent the ‘Oxford Phase-3’ medial UKA (Biomet Inc., Warsaw, Indiana, USA). The surgical technique was as recommended in the manufacturer’s surgical manual.

**Fig. 1:**
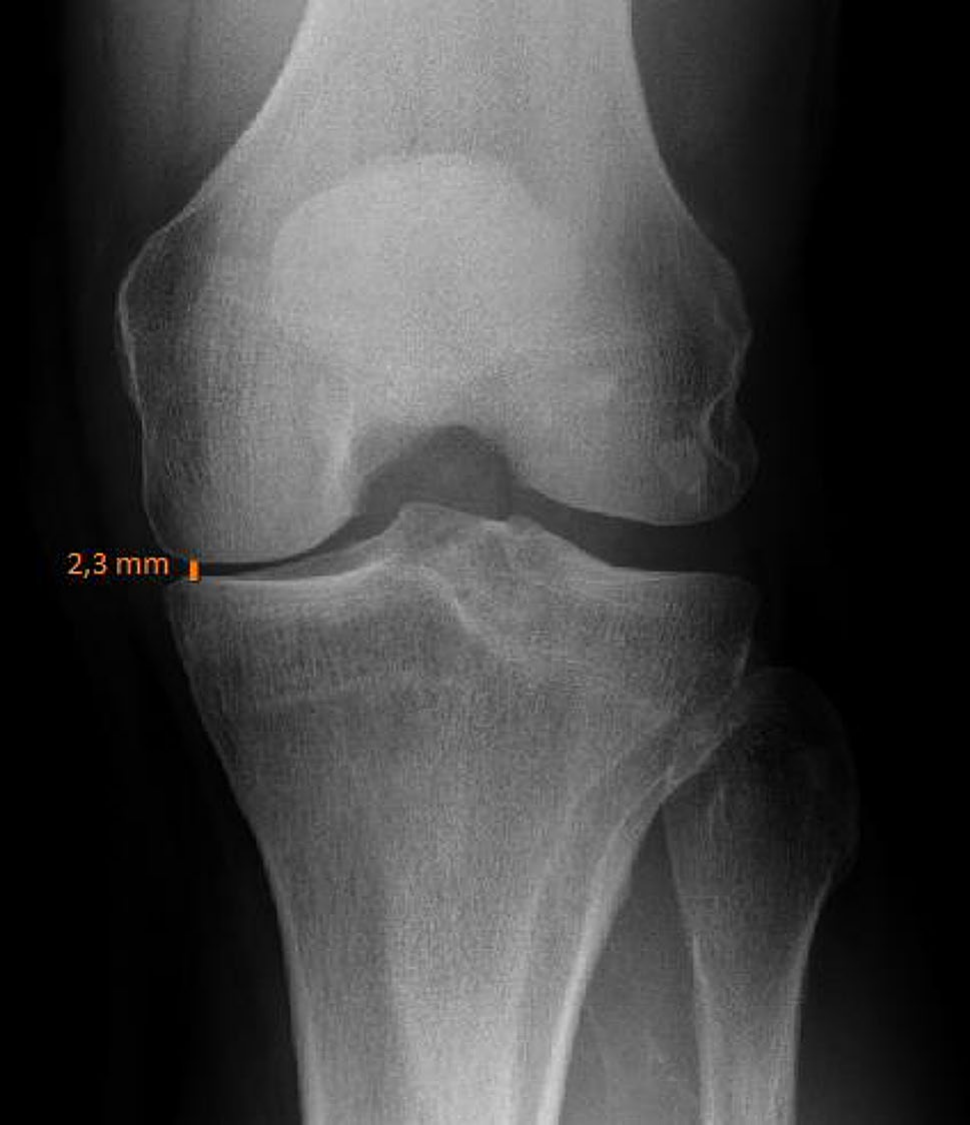
Measurement of joint space width from weight-bearing Schuss-view radiographs

Joint space width was determined from radiographs in the medical university hospital’s PACS by always the same investigator using the same software (Impax EE, Agfa Health Care N.V., Mortsel, Belgium). Among various means of radiographical determination of the severity of knee OA, previous studies recommended the measurement of joint space width due to superior reliability and validity over other methods [4, 20]. From weight-bearing flexed radiographs (Schuss-view) [8, 11, 13], the location of the most pronounced narrowing of the joint space width was identified (Fig. 1). The joint space was measured to one decimal place of a millimetre at that point to determine the parameter ‘joint space width (JSW)’. In the case of not only full joint space collapse but even bony defects (e.g. femoral condyle eroding in the tibia), JSW was defined as 0 mm because negative measurements would have been less accurate. The JSW measurements were rounded to full millimetres and patients were assigned to Group 1 if JSW was 0 or 1 mm, and to Group 2 if JSW was ≥ 2 mm.

For patient-reported outcome measurement, the Western Ontario and MacMaster Universities (WOMAC) Osteoarthritis Index score [2, 16] was analysed. The questionnaire was completed the day before surgery and again postoperatively 1 year after surgery. Implant survival as the second outcome parameter was extracted from the arthroplasty register of Tyrol.

Data analysis was performed with R version 3.6.3. (The R foundation for Statistical Computing, c/o Department of Statistics and Mathematics, University of Vienna, Vienna, Austria). The Mann–Whitney *U* test was applied to test for significant differences between groups regarding the WOMAC total score and the WOMAC subscores. For Kaplan–Meier estimation, the R package survival version 3.1.8 was used. Differences in survival curves were tested using the log rank test. Alpha was defined as 0.05 (two-tailed).

## Results

The two groups together comprised 150 patients after medial UKA (98 females, 52 males). Age was 66 years (Md, IQR 13) and BMI was 28 kg/m^2^ (Md, IQR 5.9). JSW was 1 mm (Md, IQR 1, range 0–4 mm). There were 80 patients in Group 1 (JSW 0–1 mm) (age 66, IQR 11, BMI 27.8) and 70 patients in Group 2 (JSW ≥ 2 mm) (age 64, IQR 15, BMI 28.1). In Group 2, mean JSW was 2.2 mm ± 0.4 mm (SD, range 2–4 mm). Preoperative WOMAC total and WOMAC subscores showed no significant differences between Group 1 and Group 2 (Table 1).

**Table 1:** WOMAC total scores and subscores at baseline and 1 year postoperative for the two groups

	Group 1		Group 2		*U* test
	0–1 mm JSW		≥ 2 mm JSW		
	Median	IQR	Median	IQR	*p* value
WOMAC pain preop	43	25.5	48	25.25	0.379
WOMAC stiffness preop	52.5	32.5	60	45.5	0.235
WOMAC function preop	47.3	32.9	55.3	22.9	0.133
WOMAC total preop	49.8	27.1	57.5	26.3	0.150
WOMAC pain 1y	7	16	18	46	0.047
WOMAC stiffness 1y	12.5	25	20	42.5	0.073
WOMAC function 1y	10	9.6	17	51	0.048
WOMAC total 1y	10	10	25	47	0.052

*WOMAC* Western Ontario and MacMaster Universities Osteoarthritis Index, *JSW* Joint space width, *IQR* Inter-quartile range, *preop* preoperative, *y* year, *U test* Mann–Whitney *U* test

WOMAC pain and WOMAC function exhibited significantly poorer results in patients with ≥ 2 mm JSW. WOMAC pain at 1 year postoperative was 7 ± 16 in patients with 0–1 mm JSW and 18 ± 46 in patients with ≥ 2 mm JSW (*p *= 0.047). WOMAC function at 1 year postoperative was 10 ± 9.6 in patients with 0–1 mm JSW and 17 ± 51 in patients with ≥ 2 mm JSW 1 year postoperative (*p *= 0.048, see Table 1 for full information on WOMAC data). Post hoc power analysis revealed a power of 0.91.

**Fig. 2:**
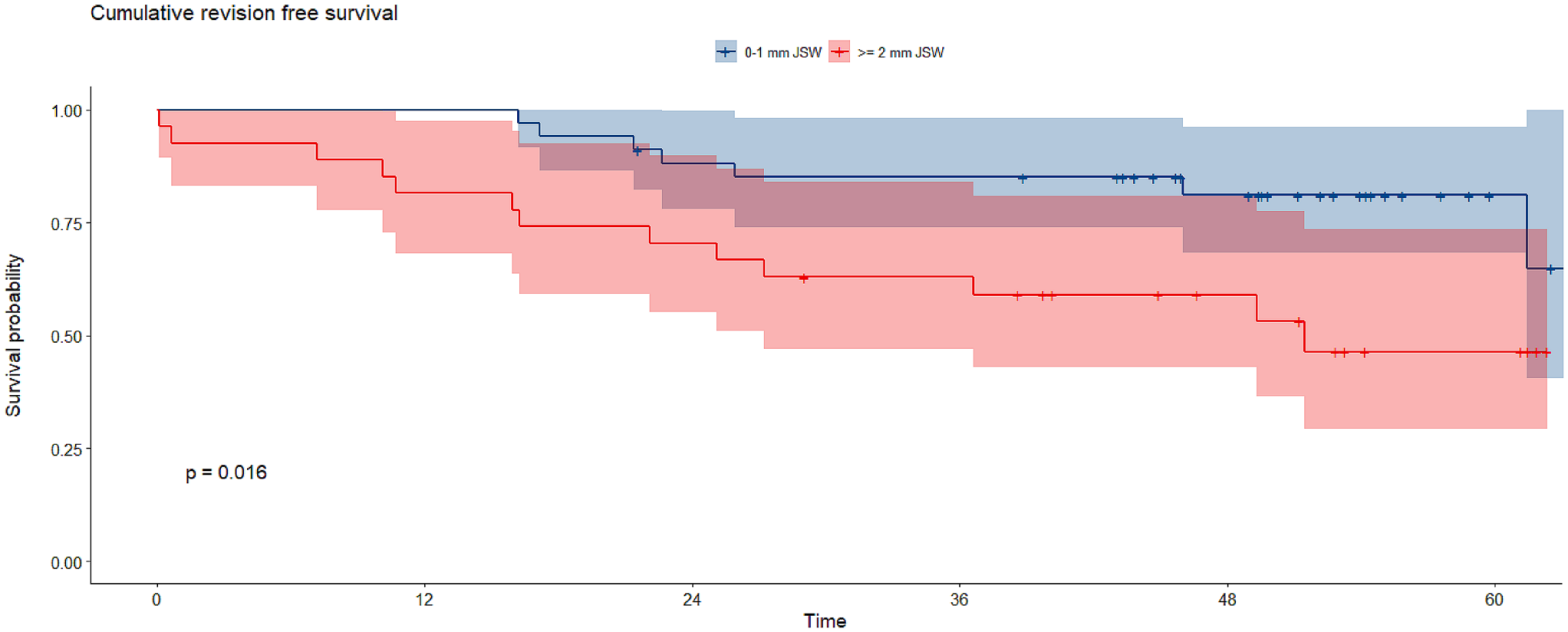
Kaplan–Meyer estimator for implant survival within 5 years postoperative for both groups

Five year prosthesis survival was 92.3% in patients with 0–1 mm JSW and 81.1% in patients with ≥ 2 mm JSW (*p *= 0.016) (Fig. 2, Table 2). Post hoc power analysis revealed a power of 0.87.

**Table 2:** Implant survival within 5 years postoperative for both groups

	Year	Survival	Upper 95% CI	Lower 95% CI
Group 1 (0–1 mm JSW)	1y	1.000	1.000	1.000
	2y	0.950	0.903	0.999
	3y	0.937	0.885	0.992
	4y	0.923	0.866	0.984
	5y	0.923	0.866	0.984
Group 2 (≥ 2 mm JSW)	1y	0.929	0.870	0.991
	2y	0.886	0.814	0.963
	3y	0.857	0.779	0.943
	4y	0.843	0.761	0.932
	5y	0.811	0.723	0.909

*JSW* joint space width, *CI* confidence interval

## Discussion

The most important findings were the significantly poorer WOMAC pain and function scores in patients in Group 2 (mean JSW 2.4 mm, range 2–4 mm), although no such significant differences were observed for the WOMAC total score. Moreover, after a 5 year follow-up interval patients with a JSW of ≥ 2 mm revealed significantly inferior implant survival as compared to patients with 0–1 mm JSW.

When attempting to compare the findings of the current study with those reported in previous publications, it was seen that only three publications are available on the identical topic [5, 7, 10]. Knifsund et al. investigated 294 UKA cases [7]. They reported that those undergoing UKA with preoperative Kellgren–Lawrence (KL) Grade 0–2 had a significantly greater risk of later reoperation than did those with KL Grade 3–4. Knifsund et al. also stated that knees with a joint space width greater than 1 mm have a greater risk for revision surgery. The findings of the current study support the findings made in the study by Knifsund et al. However, Knifsund et al. did not publish patient-reported outcome parameters, as did the current study. Therefore, no comparisons are possible in this regard. Maier et al. also investigated the influence of the preoperative stage of OA on medial UKA outcome [10]. The authors reported on 64 patients and compared cases with partial joint space collapse and those with complete joint space taken from varus stress radiographs in 20° knee flexion. Similar to the current study, Maier et al. analysed knee scores and implant survival. The authors reported that there were no significant differences between groups with regard to postoperative Oxford Knee Score, Knee Society Score or VAS pain. In addition, the authors also investigated 5 year implant survival and reported survival figures of 97% and 84% in the groups with complete and partial joint space collapse, respectively. Interestingly, the authors report a p of 0.095, which might be an indicator for a beta error, especially when interpreted together with a relatively low case number of 32 per group. In other words, it can be speculated whether Maier et al. would have achieved statistical significance if they had had more cases. Unfortunately, no power analysis was provided by Maier et al. Hamilton et al. investigated 94 UKA with preoperatively only partial cartilage loss [5]. The 94 cases were 1:2 matched with 188 cases with full-thickness cartilage loss and compared with regard to Oxford Knee Score and Knee Society Scores. After 1, 2 and 5 years, the authors found significantly poorer scores in patients with preoperatively only partial cartilage loss. In this regard, the findings made by Hamilton et al. are congruent with the findings made in the current study. Hamilton et al. also analysed implant survival and reported no differences in implant survival between UKA patients with partial cartilage loss and those with full cartilage loss (*p *= 0.06). In this connection, the findings of the current study conflict with the findings made by Hamilton et al. However, the *p* of 0.06 provided by Hamilton et al. again raises the suspicion of a beta error, especially because no power value was reported. Another potential explanation for the significantly poorer knee score results without inferior implant survival could be that Hamilton et al. were more conservative when it came to revision surgery. Interestingly, Hamilton et al. also investigated the reoperation rate (surgery without removal of any of the UKA components). In this connection, the authors found highly significant differences between the groups with a 5 year reoperation rate of 10.9% and 3.9% for patients with partial cartilage loss and full cartilage loss, respectively (*p *< 0.001).

When attempting to analyse for differences amongst the three previous studies, it is seen that only two studies investigated knee scores [5, 10]. Of these two, only one study found a significantly poorer score outcome among patients with incomplete joint space collapse [5]. All three previous studies investigated implant survival. Only one of them found significantly poorer implant survival among patients with incomplete joint space collapse [7], while the other two reported a p between 0.05 and 0.1, and therefore, potentially suffered from beta error [5, 10], especially because no power was stated.

In the TKA field, the situation seems to be clearer. Seven studies were published that examined the relationship between preoperative OA severity and clinical outcome [6, 9, 12, 14, 17–19]. In summary, the results favoured an association between preoperative OA grade and TKA outcome (five studies pro: two studies contra: the more preoperative degeneration, the better the TKA outcome. However, the TKA field does not support clear conclusions for UKA patients.

The following study limitations are acknowledged. First, it was a retrospective study with the typical weaknesses associated with such studies: selection bias, information bias, inability to investigate parameters other than those previously collected during clinical routine, reliance on data collected by others etc. Second, although previously suggested we did not succeed in collecting physical activity data or health-related quality of life data in conjunction with the knee-specific WOMAC data. Third, it was not possible to control for proper stratification of preoperative symptoms. As this was a retrospective study, it was just pure coincidence that the preoperative WOMAC scores were balanced between the groups. As the groups showed no differences in terms of WOMAC prior to surgery, this means surgical indication was made predominantly on the basis of clinical and subjective assessment and not radiography.

It is regarded as a strength of our study that radiographic severity of knee OA was assessed in terms of JSW from weight-bearing radiographs, which was found to be the preferable method [4, 20]. Another strength was that both a well-known outcome parameter (WOMAC) and the implant survival rate were investigated.

The study findings are regarded as having high clinical relevance. Particularly high patient satisfaction can be expected when using‚ complete joint space collapse’ as indication for UKA surgery. In the case of incomplete joint space collapse, further conservative therapy [1, 3] or joint-preserving knee surgery (guided by MRI diagnostics) [15] might be considered an alternative to UKA.

## Conclusions

Clinical outcome in patients with preoperative complete joint space collapse (0–1 mm JSW) was superior to that in patients with incomplete joint space collapse. This was true for both 1 year postoperative WOMAC pain and WOMAC function and for 5 year implant survival rates. Complete radiographic joint space collapse provides superior outcome after UKA as compared with partial joint space collapse.

## Abbreviations

JSW: Joint space width
UKA: Unicondylar knee arthroplasty
WOMAC Score: Western Ontario and MacMaster Universities Osteoarthritis Index Score
BMI: Body mass index
TKA: Total knee arthroplasty
KL: Kellgren–Lawrence
OA: Osteoarthritis
PACS: Picture Archiving and Communication System
